# Numerical Study of the Microflow Characteristics in a V-ball Valve

**DOI:** 10.3390/mi12020155

**Published:** 2021-02-04

**Authors:** Zhi-xin Gao, Yang Yue, Jia-ming Yang, Jun-ye Li, Hui Wu, Zhi-jiang Jin

**Affiliations:** 1 SUFA Technology Industry Co., Ltd, CNNC, Suzhou 215129, China; yueyang@chinasufa.com (Y.Y.); lijy@chinasufa.com (J.-y.L.); wuh@chinasufa.com (H.W.); 2 Institute of Process Equipment, College of Energy Engineering, Zhejiang University, Hangzhou 310027, China; yangjiaming@zju.edu.cn (J.-m.Y.); jzj@zju.edu.cn (Z.-j.J.)

**Keywords:** control valve, microflow, flow coefficient, loss coefficient, computational fluid dynamics

## Abstract

V-ball valves are widely applied in many process industries to regulate fluid flow, and they have advantages of good approximately equal percentage flow characteristics and easy maintenance. However, in some applications, the V-ball valve needs to have good performance under both large and extremely small flow coefficients. In this paper, the improvement of the original V-ball valve is made and the flow characteristics between the original and the improved V-ball valve are compared. Two types of small gaps are added to the original V-ball, namely the gap with an approximately rectangular port and the gap with an approximately triangular port. The effects of the structure and the dimension of the gap on flow characteristics are investigated. Results show that within the gap, the flow coefficient increases but the loss coefficient decreases as the valve opening increases, and the flow coefficient has an approximately linear relationship with the flow cross-area of the added gap. Results also show that under the same flow cross-area, the flow coefficient has a higher value if the distance between the gap and the ball center is greater or if the gap is an approximately rectangular port, while the loss coefficient has an opposite trend.

## 1. Introduction

Precise flow control is needed during fluid transportation in many process industries, including the nuclear power station, thermal power station, and petrochemical industries. Control valves, which can provide flow or pressure control, are often applied in these industries and normally have good performance. Based on the movement direction, control valves can be divided into two types, namely the linear motion control valves and the rotary motion control valves. A typical linear motion control valve is the globe valve, and a typical rotary control valve is the butterfly valve; the fluid flow control is mainly achieved by the adjustment of the flow cross-area inside the control valves.

In the past few decades, various types of control valves have had rapid development, and many works focusing on control valves have been done. In the beginning, experiments were the main method applied, but with the development of computational fluid dynamics (CFD) and computer technology, numerical simulations have become the popular method. Vibration, cavitation, and noise are the main problems that a control valve may suffer, and they have attracted a lot of attention. Yonezawa et al. [1] found that the valve head vibration is caused by the unsteady flow around the control valve. Zeng et al. [2] found that the process of the pressure ratio variation affects the valve vibration and noise fluctuations in a control valve. Li et al. [3] found that the inlet pressure fluctuation is the necessary and sufficient condition to yield force vibration. Jin et al. [4,5] investigated the effects of the valve core shapes and valve body structure on cavitation intensity inside a sleeve regulating valve and a globe valve. Li et al. [6] studied the cavitation suppression method in a globe valve with a valve cage. Li et al. [7] and Qian et al. [8] focused on the cavitation inside a mechanical heart valve. Sreekala and Thirumalini [9] found that the sound pressure level could be reduced by adding the flow passage numbers in the valve cage of a globe valve. Qian et al. [10,11] investigated the aerodynamic noise in a temperature and pressure regulation valve and a Tesla valve.

The flow characteristics [12,13,14,15,16,17,18,19] and dynamic characteristics [20,21,22] of a valve are the key indicators for judging the performance of the valve in a specific application. For the flow characteristics, research work has mainly focused on the flow coefficient. Qian et al. [12] compared four kinds of pilot-control globe valves in terms of the flow characteristics and found that the valve with a parallel mounted pipe had better hydro-performance. Qian et al. [13,14] also investigated the effects of the plug shape in a globe valve [13] and the effects of the shape of the throttling window in a sleeve globe valve [14] on the intrinsic flow characteristics, and they proposed equations used to calculate the outlet flow rate [13] and the valve flow characteristics [14]. Nguyen et al. [15] experimentally tested the flow and pressure distributions in a globe valve, and their results showed that the valve flow coefficient was not only affected by the valve dimension but also affected by the Reynolds number. Singh et al. [16] compared the effects of the manufacturing method, including the selective laser melting method and the electron discharge machining method of trim cages in a control valve on the valve characteristics, and they found that the valve trim obtained by the selective laser melting method had a higher surface roughness, which led to the increase of the wall shear stress and the decrease of the flow capacity. Iravani and Toghraie [17] experimentally tested the flow characteristics of a ball valve in a compressible flow, and their results showed that the flow coefficient and loss coefficient were affected by the pressure and the flow rate and the loss coefficient had an inverse relationship with the pressure. Asim et al. [18] focused on a valve trim made of a staggered arrangement of columns in a control valve, and they investigated the effects of the size of the flow path on the flow capacity and found a critical flow path size. Chern et al. [19] numerically investigated the relationship between the flow coefficient and the flow cross-sectional area of the passages in a single cage and in multicages to help future valve cage design. For the dynamic characteristics, research work has mainly focused on the movement of the valve trim. Gao et al. [23] compared the dynamic characteristics of two types of check valves in a compressible fluid. Lisowski et al. [24] investigated the flow force acting on the spool during the initial opening phase of the spool gap. Qiu et al. [25] investigated the effects of valve needle speed on flow characteristics, concluding that high valve needle speed leads to a large flow rate under large valve openings.

The V-ball valve is an improved valve based on the traditional ball valve, and it can provide better flow characteristics in various fluids like water, oil, or an oxidizing medium. Compared with other fluid control valves, the V-ball valve is more easily maintained and replaced once there is valve damage. At this time, little work can be found focusing on the V-ball valve. Chern and Wang [26] investigated the flow and loss coefficients of a V-port valve under different valve openings. Zhang et al. [27] proposed a structural improvement of the V-ball to create ideal equal percentage flow characteristics, while the adjustment of a small flow coefficient under small valve openings still had a bad performance. Tao et al. [28] investigated the flow and loss coefficients with a different V-angle of the V-ball. They proposed a fitting equation between the flow coefficient and the valve opening and the V-angle, but the valve diameter was not considered, limiting the proposed equation.

In some applications, such as in nuclear industries, the utilized control valve needs to supply both a large flow coefficient on the order of 10 and a small flow coefficient on the order of 10^−1^, so the traditional V-ball valve needs to be improved, especially for small valve openings. In this paper, a computational fluid dynamics method is used to study the flow characteristics of a V-ball valve. Firstly, improvement of the V-ball is made to achieve the adjustability of a small flow coefficient, and then the flow and loss coefficients of the improved V-ball valve under small valve openings are calculated. Finally, the flow cross-area is calculated, and the relationship between the flow and loss coefficients and the flow cross-area is obtained.

## 2. Methods 

### 2.1. Physical Model

**Figure 1:**
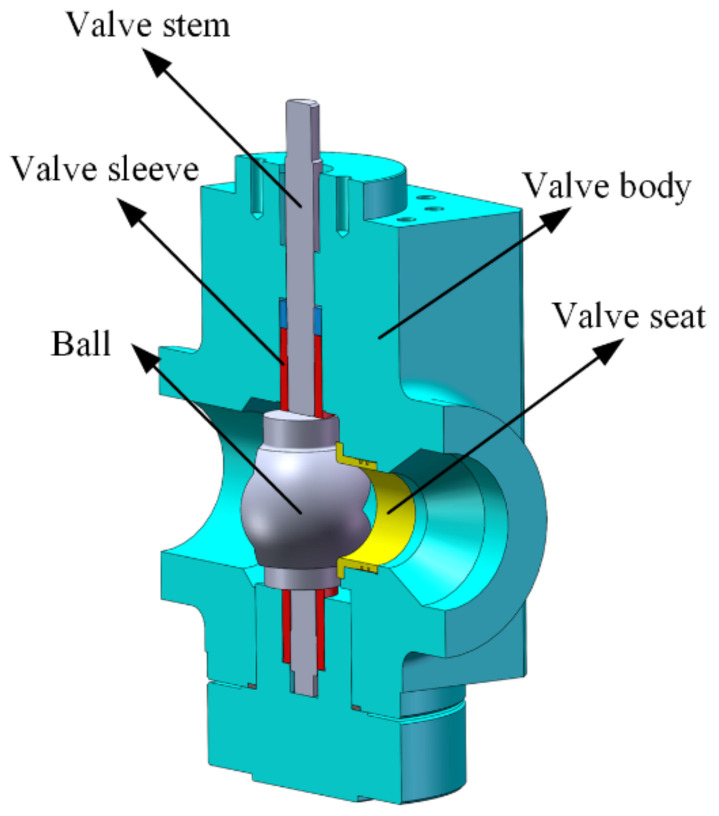
Structure of the V-ball valve.

The original V-ball valve mainly consists of the valve seat, the valve body, the valve stem, the valve sleeve, and the ball. An approximately triangular flow cross-section was machined on the ball as shown in Figure 1.

The dimensions of the investigated V-ball valve are listed as follows: The nominal diameter of the upstream and downstream pipes, *D*, was 125 mm, and the diameter of the valve seat was 70 mm. The length of the upstream pipe was 5*D*, and the length of the downstream pipe was 8*D*.

### 2.2. Numerical Model

Water under room temperature was considered as the working fluid, and the heat transfer was ignored during the numerical simulations. To obtain the flow inside the investigated V-ball valve, the continuity and the momentum equations need to be solved; relative equations are shown as follows:

(1) $$\frac{\partial }{\partial x_{j}}u_{j}=0$$

(2) $$\frac{\partial }{\partial x_{j}}\left(\rho u_{i}u_{j}+p\delta_{ij}-\tau_{ij}\right)=0$$

Because this study focuses on the microflow characteristics, the Reynolds number within the main valve was set at a low level to avoid cavitation, which means the SST *k-ω* turbulence model is more applicable compared with the *k-ε* turbulence model, because the *k-ε* turbulence model is often applied with a high Reynolds number. The relevant transport equations of the turbulence model are shown below.

(3) $$\frac{\partial }{\partial x_{i}}\left(\rho ku_{i}\right)=\frac{\partial }{\partial x_{j}}\left(\Gamma_{k}\frac{\partial k}{\partial x_{j}}\right)+G_{k}-Y_{k}$$

(4) $$\frac{\partial }{\partial x_{i}}\left(\rho \omega u_{i}\right)=\frac{\partial }{\partial x_{j}}\left(\Gamma_{\omega }\frac{\partial \omega }{\partial x_{j}}\right)+G_{\xi }-Y_{\omega }+D_{\omega }$$

Here *ρ* stands for the water density, *u* stands for the velocity in each grid, *p* stands for the pressure, *τ*_ij_ stands for the viscous stress, *G_k_* and *G_ω_* stands for the generation of turbulent kinetic energy and specified dissipation rate, *Г* stands for the effective diffusivity, *Y_k_* stands for the dissipation caused by turbulence, and *D_ω_* stands for the cross-diffusion term.

Commercial software ANSYS Fluent based on the finite volume method was used to solve the governing equations described above. The semi-implicit method for pressure-linked equations (SIMPLE) algorithm and the second-order upwind discretization method were utilized. The residuals were set as 10^−3^, and the inlet velocity was monitored. Solutions were considered as converged if all residuals were below 10^−3^ and the monitored inlet velocity was stable. Pressure inlet, pressure outlet, and no-slip wall were set as the boundary conditions. The inlet pressure was 16.08 MPa, the outlet pressure was 15.48 MPa, and the wall roughness was not considered. Wall y+ lay between 0 and 100 for different cases of improved V-ball valves.

**Figure 2:**
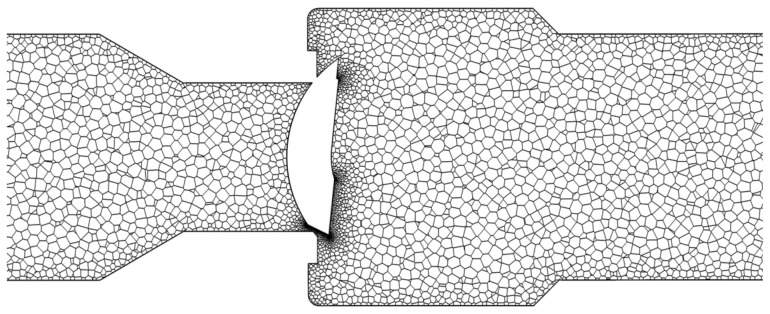
Mesh of the improved V-ball valve.

A polyhedral mesh with boundary layers of the improved V-ball valve is shown in Figure 2. Grid independence was implemented to eliminate the influence of the grid number under a small valve opening, and the flow rate of the investigated V-ball valve under the different grid number is shown in Table 1, where the benchmark value used to obtain the difference of the flow rate is the flow rate under grid #2. It shows that when the grid number is 9.16×10^5^, the difference of the flow rate between different grid number is very small, so the method used to generate grid #2 was adopted.

**Figure 3:**
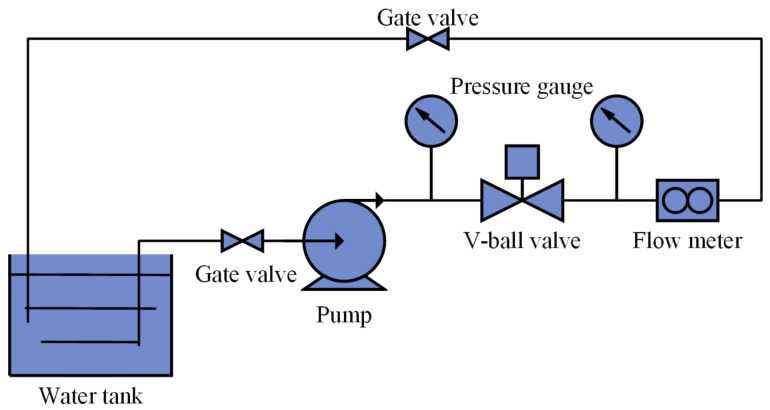
The sketch of the test rig.

**Figure 4:**
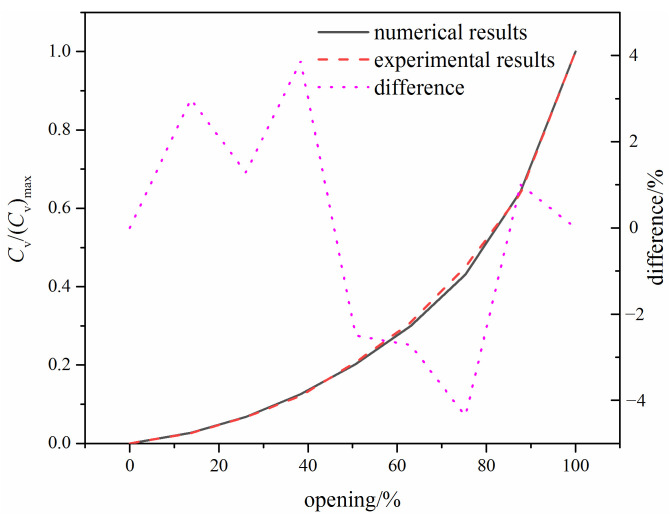
Comparison between the experimental results and the numerical results.

To validate the adopted numerical methods, experimental data of the flow coefficient for the V-ball valve shown in Figure 1 were obtained using a specific flow coefficient test rig. The sketch of the test rig and the comparison between the numerical and the experimental results are shown in Figure 3 and Figure 4, respectively. It can be found that the maximum difference between the experimental results and the numerical results is less than 5%, and thus the numerical methods adopted in this study are effective.

### 2.3. Analysis Method

For a control valve, the flow characteristics are often used to judge whether the control valve has good performance in actual operating conditions. Normally, the minimum flow coefficient and the maximum flow coefficient should be within valve openings ranging from 10% to 70%. In this study, the flow coefficient is calculated, compared, and analyzed, and the expression of the flow coefficient, *C_v_*, is shown as Equation (5) if the Reynolds number of the V-ball valve, *Re_v_*, is above 10,000 [29].

(5) $$C_{v}=11.56\cdot Q\cdot \sqrt{\frac{\rho /\rho_{0}}{\Delta p}}$$

(6) Rev=N4FdQνCvFLFL2Cv2N2D4+11/4

Here, *Q* stands for the flow rate (m^3^/h), *ρ*_0_ stands for the water density at 15°, *∆p* stands for the pressure difference between the inlet and the outlet of the V-ball valve, *F_d_* is a correction factor, *F_L_* is the pressure recovery coefficient, ν stands for the kinematic viscosity, and *N_2_* and *N_4_* are model constants. In this study, *F_d_* is set as 0.46, *F_L_* is set as 0.9, *N_2_* is 0.00214, and *N_4_* is 0.076.

## 3. Results

This section first presents the flow characteristics of the original V-ball valve and then compares them with those of the improved V-ball valve. Secondly, the flow and loss coefficients under small valve openings are presented and analyzed. Finally, results are presented for the calculation of the flow cross-area of the millimeter-scaled channel on the improved V-ball is calculated, and the correlation between the flow cross-area and the flow and loss coefficients is presented.

### 3.1. Flow Coefficient

**Figure 5:**
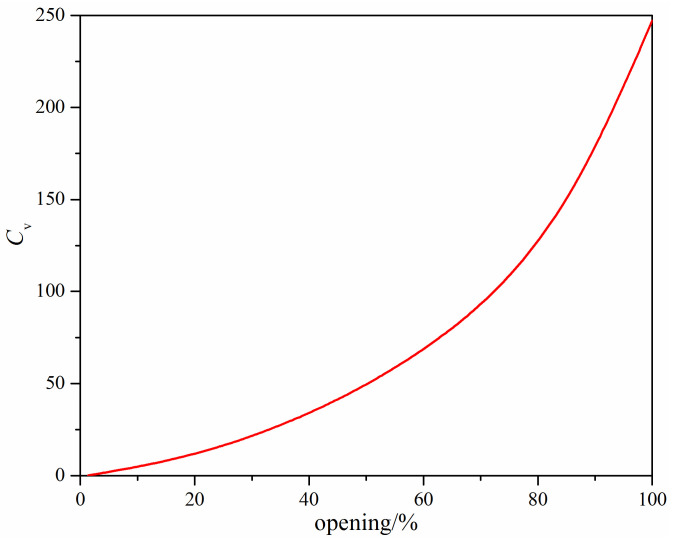
Flow characteristics of the original V-ball valve.

For the original V-ball described in Figure 1, the flow characteristic is shown in Figure 5. It shows that the flow characteristic is similar to the ideal equal percentage flow characteristic, which is similar to the results obtained by Tao et al. [28]. Figure 5 also shows that when the flow coefficient is on the order of 10^−1^, the relative valve opening is around 1%. However, because of the delay of the valve regulation, the V-ball valve may be closed during the operation, which leads to the poor performance of the valve.

**Figure 6:**
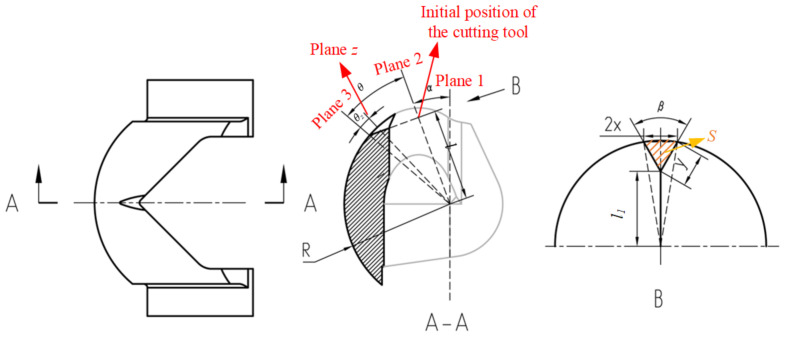
Improvement #1 of the V-ball.

To meet the demand for a wide range of flow coefficient and long-term low flow rate, an improvement was made to the V-ball. The main problem of the original V-ball valve is that the flow cross-area under small valve openings is too large to fulfill the demand of an extremely small flow coefficient. Thus, a small gap can be added on the V-ball to achieve a small flow cross-area. As shown in Figure 6, an additional small gap was added by cutting the V-ball using a tool with a V-shape head. Here, the distance between the head tip of the tool and the center of the V-ball is *l*; the cutting tool with a *β* degree V-shape head on plane 1 firstly rotates *α* degrees, then cuts the ball in the perpendicular direction of plane 2.

**Figure 7:**
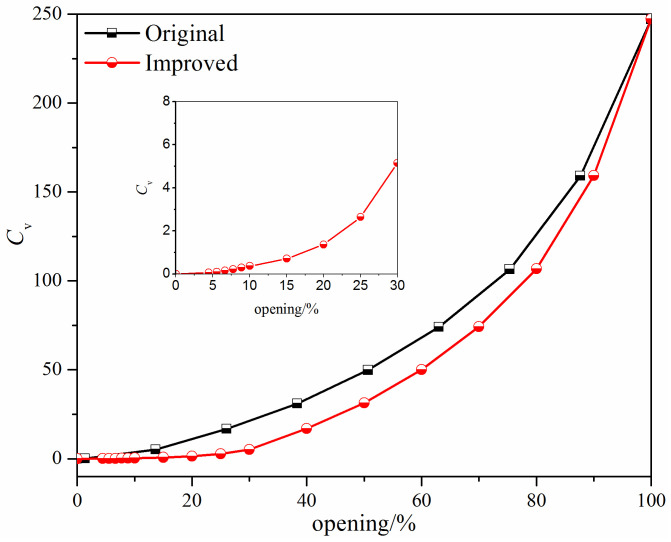
The flow characteristics of the improved V-ball valve.

The flow characteristics of the improved V-ball valve when *α* is 20°, *β* is 60°, *l* is 48 mm, and the radius of the V-ball, *R*, is 56 mm are shown in Figure 7. This clearly shows that the adjustability is better for the improved V-ball valve.

**Figure 8:**
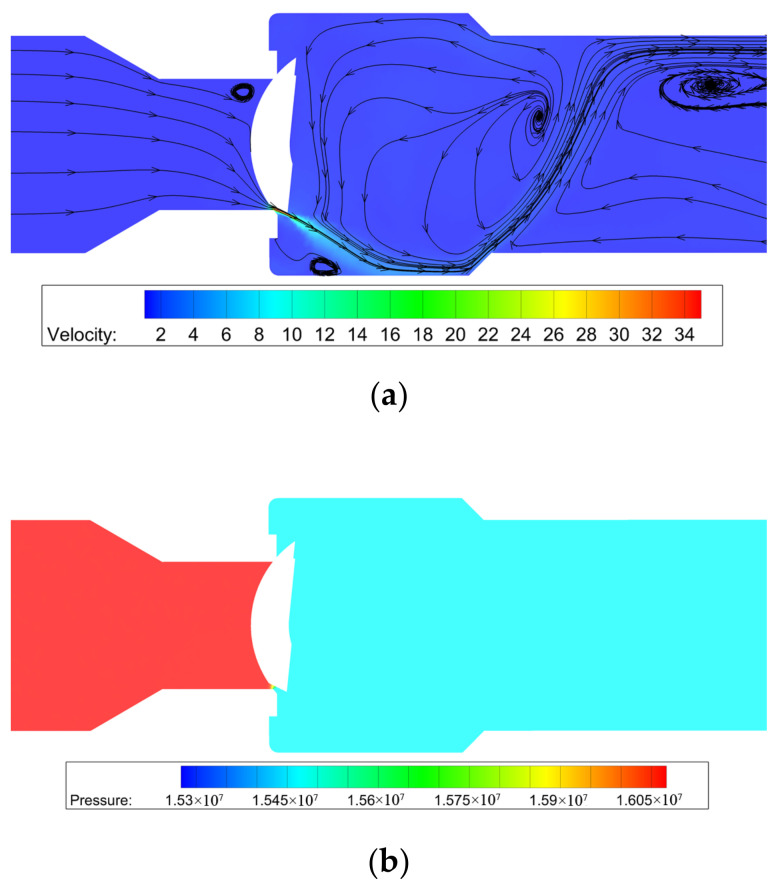
The velocity and pressure distributions of the improved #1 V-ball valve: (**a**) velocity and streamline distributions; (**b**) pressure distribution.

The velocity and pressure distributions of the improved #1 V-ball valve are shown in Figure 8, where the valve opening is around 7%. It can be found that by adding a gap, the decompression ability of the V-ball valve is increased, so the flow coefficient can be decreased. Moreover, a large velocity appears in the gap, which means the material of the ball needs to be treated appropriately.

**Figure 9:**
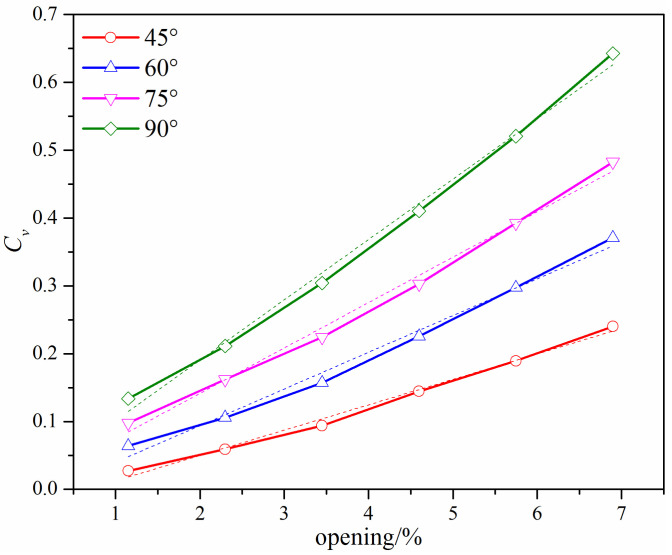
The flow coefficient of the improved V-ball valve with different *β* degrees when *l* = 48 mm.

To investigate the effects of the dimension of the added gap on the flow characteristics, the flow coefficient under small valve openings formed by the gap and the valve seat were simulated and compared. When *l* is 48 mm, the flow coefficient under different valve openings with different *β* degrees is shown in Figure 9. It can be found that the flow coefficient increases with the increase of *β* degree under the same valve opening. The dashed lines in Figure 9 are the linear fitting results based on the simulated data. A comparison of the fitted results and the computed results shows that the relationship between the flow coefficient and the valve opening is similar to the linear flow characteristics under small valve openings; the lower the *β* degree, the smaller the deviation between the fitted results and the simulated results. The approximately linear relationship means that when a small V-shape gap is added on the V-ball, the V-ball valve has a linear flow characteristic under small valve openings but equal percentage flow characteristic under large valve openings. Moreover, the slope of the fitted curve increases with the increase of *β* degree, indicating that the variation of the flow coefficient under different valve openings increases as the *β* degree increases.

**Figure 10:**
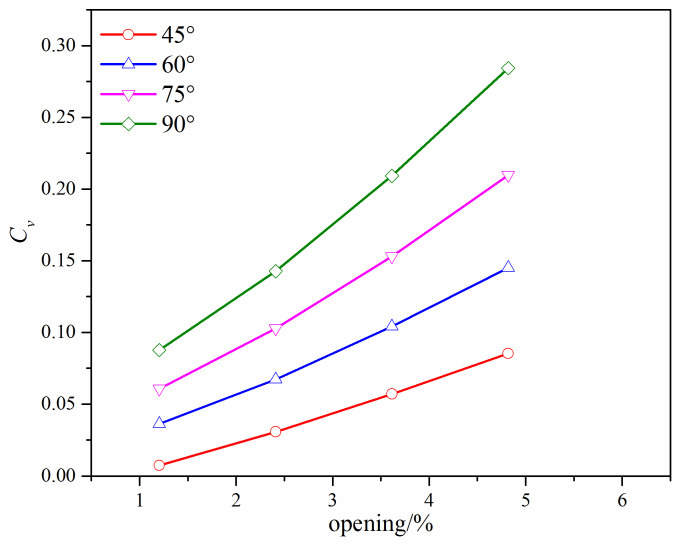
The flow coefficient of the improved V-ball valve with different *β* degrees when *l* = 50 mm.

When *l* is 50 mm, the flow coefficient under different valve openings with different *β* degrees is shown in Figure 10, where only the valve opening less than 5% is focused on because the added gap is not the only flow channel once the valve opening is larger than 5%. When compared with Figure 9, the distance *l* does not affect the variation trend of the flow coefficient, and the flow coefficient increases with the *β* degree increase in an approximately linear way. In Figure 10, it can be seen that the flow coefficient decreases as the distance *l* increases, which results from the decrease of the flow cross-area.

**Figure 11:**
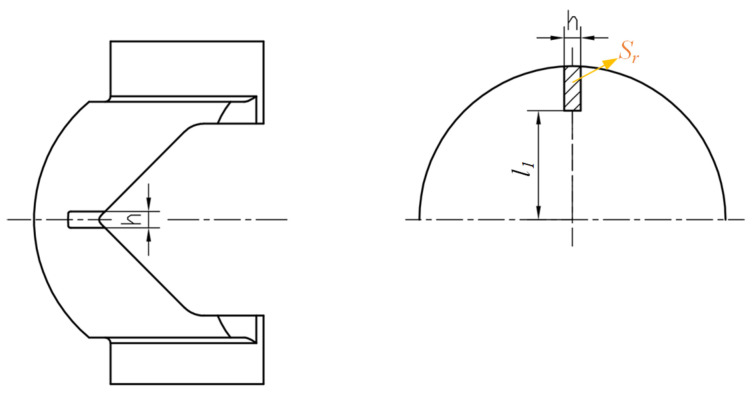
Improvement #2 of the V-ball.

If the head of the cutting tool has a rectangular lateral surface, the formed gap on the V-ball can have a larger flow cross-area. The structure of the improved #2 V-ball is shown in Figure 11. 

**Figure 12:**
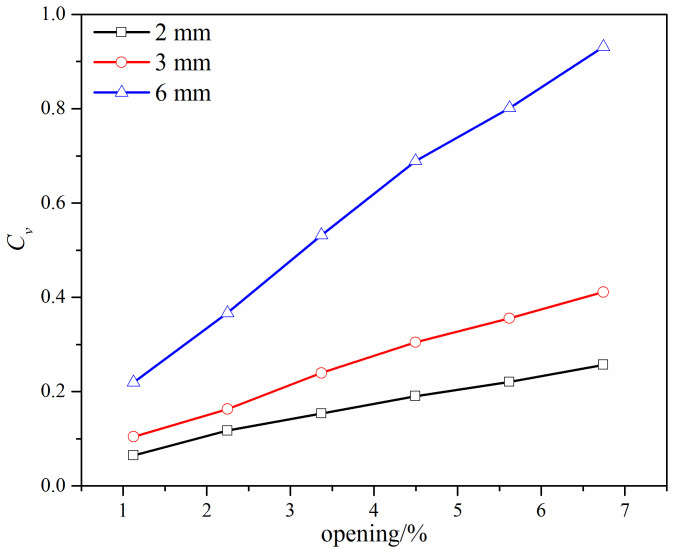
The flow coefficient of the improved V-ball valve with different *h* values when *l* = 50 mm.

Assuming that the width of the gap is *h*, when *l* is 48 mm, the flow coefficient under different valve openings with different *h* values is shown in Figure 12. Figure 12 shows that the flow coefficient increases with the increase of the width *h*, as the flow cross-area increases with the increase of the width *h*. It also shows that the relationship between the flow coefficient and the valve opening is also approximately linear, and the slope increases as the width *h* increases. Considering these results along with those shown in Figure 9, Figure 10, and Figure 11, it can be concluded that, under small valve openings, the relationship between the flow coefficient and the valve opening always tend to be approximately linear and will not be affected by the structure and the dimension of the added gap on the V-ball.

To compare the flow characteristics of the improved V-ball valve with different structures of the gap directly, the flow coefficient under different flow cross-areas and different structures of gaps were analyzed. For improvement #1 of the V-ball, as shown in Figure 6, *R* stands for the radius of the V-ball, and plane 3 is the end of the added gap. Assume the valve opening is *z*, and the relative angle of rotation is *θ_z_*. On plane *z*, assume the distance between the tip of the gap and the ball center is *l*_1_, the distance between the tip of the gap and the wall of the ball is *y*, and the chord length formed by the cutting tool is 2*x*, then the flow area on plane *z*, *S*, which is shown in Figure 6 can be approximately expressed as follows:

(7) $$S=R^{2}\cdot \mathrm{acrsin}\frac{x}{R}-x\cdot R\cdot \cos\left(\mathrm{acrsin}\frac{x}{R}\right)+x\cdot \left(\sqrt{R^{2}-x^{2}}-l_{1}\right)$$

Here,

(8) $$l_{1}^{2}+y^{2}-2\cdot l_{1}\cdot y\cdot \cos\left(\left(180-\frac{\beta }{2}\right)\cdot \frac{\pi }{180}\right)-R^{2}=0$$

(9) $${\left(\sqrt{R^{2}-x^{2}}-l_{1}\right)}^{2}+x^{2}-y^{2}=0$$

(10) $$l_{1}=\frac{l}{\cos\left(\left(\theta -\theta_{z}\right)\cdot \frac{\pi }{180}\right)}$$

For improvement #2 of V-ball, the flow area on plane *z*, *S_r_*, which is shown in Figure 11 can be approximately expressed as follows:

(11) $$S_{r}=R^{2}\cdot \arcsin\frac{h}{2R}-\frac{h}{2}\cdot R\cdot \cos\left(\arcsin\frac{h}{2R}\right)+h\cdot \left(\sqrt{R^{2}-{\left(h/2\right)}^{2}}-l_{1}\right)$$

**Figure 13:**
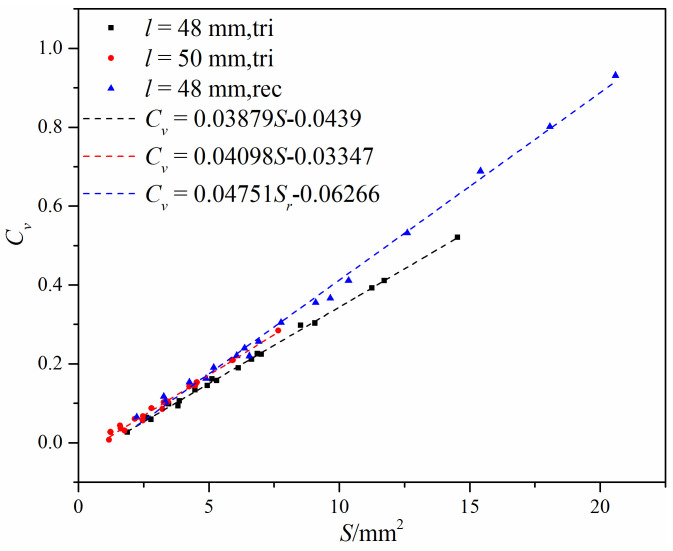
The flow coefficient under different flow cross-areas and different gap structures.

The analyzed results of the simulated flow coefficient and the calculated flow cross-area are shown in Figure 13. It can be seen from Figure 13 that the relationship between the flow coefficient and the flow cross-area can be regarded as approximately linear, except that there is a large fluctuation when the flow cross-area is less than 3 mm^2^. With the increase of distance *l*, the flow coefficient increases under the same flow cross-area. Under the same distance *l* and the same flow cross-area, the flow coefficient is also affected by the gap structure, and the gap with an approximately rectangular port has higher flow capacity than the gap with an approximately triangular port. Moreover, it shows that a larger flow cross-area results in greater effects of the distance *l* and the gap structure.

### 3.2. Loss Coefficient

The loss coefficient, *ξ*, is expressed as follows:

(12) $$\xi =\frac{2\Delta p}{\rho v^{2}}$$

**Figure 14:**
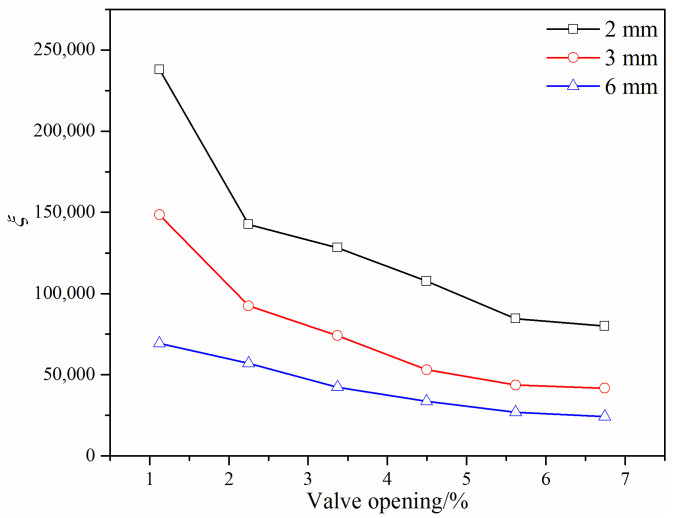
Loss coefficient of the improved #2 V-ball valve under different valve openings.

Here *v* is the mean velocity inside the V-ball valve. For a given gap structure and a fixed distance *l*, the loss coefficient should decrease with the increasing valve opening, because the loss coefficient has an inversely proportional relationship with the square of the flow coefficient. Figure 14 shows the loss coefficient under different small valve openings and different widths for the improved #2 V-ball valve. It can be found that the loss coefficient decreases as the valve opening increases. With the increase of the width *h*, the loss coefficient decreases, and the variation of the loss coefficient increases with the decrease of the width *h*.

**Figure 15:**
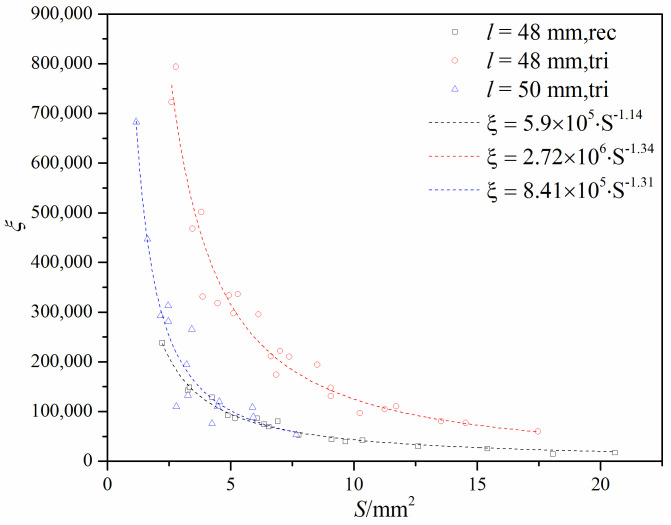
Loss coefficient under different flow cross-areas and different gap structures.

Figure 15 shows the loss coefficient under different flow cross-areas. It is found that the loss coefficient is affected by the gap structure and the distance *l* for the same flow cross-area. When there is the same gap structure, the obvious difference can be found under different distance *l*, while there is the same distance *l*, the difference of the loss coefficient under different gap structure is not obvious. Figure 15 also shows that the relationship between the loss coefficient and the flow cross-area is not linear, and its correlation can be expressed as *ξ* = *a*∙*S^b^*, where *a* and *b* are model constants (values shown in Figure 15).

## 4. Conclusions

To improve the adjustability of normal V-ball valves in some specific working conditions, where both a large flow coefficient and an extremely low flow coefficient are needed, an additional small gap was introduced to the V-ball. In this study, the computational fluid dynamics method was used to compare the performance of the improved and original V-ball valves and study the effects of the introduced gap structure on the flow characteristics. 

Two gap structures formed by a cutting tool with a V-shape head or a head with a rectangular lateral surface, which are called the gap with an approximately triangular port and the gap with an approximately rectangular port, were investigated. For both gap structures, the flow coefficient increased and the loss coefficient decreased with the increase of the valve opening. The flow coefficient increased with the increase of the *β* degree or the width *h*, while the loss coefficient had the opposite trend. The flow coefficient had an approximately linear relationship with the valve opening under small valve openings, regardless of the gap structure.

The flow coefficient had an approximately linear relationship with the flow cross-area under the same gap structure and the same distance *l*. Under the same flow cross-area and the same distance *l*, the V-ball valve with an approximately rectangular port gap had a higher flow coefficient. Under the same flow cross-area and the same gap structure, the V-ball valve with a gap of larger distance *l* had a higher flow coefficient. The loss coefficient had a nonlinear relationship with the flow cross-area and could be expressed in the form of *ξ* = *a*∙*S^b^*.

This study examined the relationship between the flow and loss coefficients and the flow cross-area of the gap, but the relationship between the nondimensionalized flow cross-area and the flow coefficient could be more helpful and will be the subject of future work. This study provides constructive ideas for solving similar problems including the design of control valves for different purposes.

## Figures and Tables

**Table 1:** Flow rate of the improved V-ball valve with the different grid numbers.

Grid Set	Grid Number × 10^5^	Flow Rate/m^3^h^−1^	Difference/%
#1	5.59	0.378	7.1
#2	9.16	0.353	-
#3	21.22	0.363	2.8
